# Rhizobacterial diversity, community composition, and the influence of keystone taxa on O’Neal blueberry (*Vaccinium corymbosum*)

**DOI:** 10.3389/fmicb.2024.1460067

**Published:** 2024-09-13

**Authors:** Mingyun Jia, Zhuangzhuang Liu, Jiguang Wei, Qi Li, Zhaoqi Hou, Ling Sun, Hong Yu, Jinping Yu, Shipeng Lu

**Affiliations:** ^1^Institute of Botany, Jiangsu Province and Chinese Academy of Sciences, Nanjing, China; ^2^Jiangsu Key Laboratory for the Research and Utilization of Plant Resources, Nanjing, China; ^3^Nanjing Botanical Garden Mem. Sun Yat-Sen, Nanjing, China; ^4^College of Resources and Environmental Sciences, Nanjing Forestry University, Nanjing, China

**Keywords:** blueberry, rhizobacterial diversity, microbial community composition, *Bacillus*, *Vicinamibacterales*

## Abstract

Rhizosphere microbiotas play vital roles in resisting environmental stress, transforming soil nutrients, and promoting plant health, growth, and productivity. The effects of rhizosphere microbial community shaping and the characteristics and functions of keystone taxa on blueberries were comprehensively studied by examining the rhizobacteria of healthy old trees (O), young seedlings (OG), and poorly growing seedlings (OB) of O’Neal blueberries. Our results showed that rhizobacterial diversity followed the order OB > > OG > O, and the microbial community of OG was similar to that of O, while that of OB was distinctly different. The predominant rhizobacteria identified included *Actinobacteria*, *Proteobacteria*, *Firmicutes*, *Chloroflexi*, and *Acidobacteria*. *Firmicutes* were highly enriched in healthy blueberries, with *Bacillus* identified as a key genus that significantly enhanced blueberry growth when inoculated. *Bradyrhizobium* and *Gaiellales* were common core bacteria in the blueberry rhizosphere. In contrast, *Acidobacteria* were the predominant phylum in poorly growing OB, with the specific *Vicinamibacterales*-related and *Latescibacterota*-related genera acting as keystone taxa that shaped the microbial community. In addition, bacterial species in *Vicinamibacterales* might act as a potential pathogen predicted by BugBase. Taken together, these findings provide fundamental insights into the development of the blueberry rhizosphere microbial community and highlight the role of beneficial rhizobacteria, such as *Bacillus*, in enhancing blueberry growth. This knowledge could contribute to the exploitation of beneficial rhizobacteria and the prevention of pathogens in modern agriculture.

## 1 Introduction

Blueberry (*Vaccinium corymbosum*) is considered a functional food and is attracting more and more attention in the world (Yang W. et al., 2022). Its fruit contains high contents of anthocyanin, polyphenols, and flavonoids (Kalt et al., 2001; Basu et al., 2010; Yang W. et al., 2022) which have antioxidant activity and are beneficial to human health by lowering blood pressure, controlling obesity, preventing the occurrence of cardiovascular diseases, and managing type 2 diabetes (Kalt et al., 2020; Delpino et al., 2022). Due to their nutritional value and health benefits, blueberries are becoming increasingly popular with consumers worldwide. The global cultivation area of blueberries increased by 34.14% from 2012 to 2016 (Gallegos-Cedillo et al., 2018) and has been increasing continuously. China is the fastest-growing country in the blueberry industry. The cultivation area reached 66,400 ha in 2020, being the largest cultivation area in Asia (Yu et al., 2020). Yet the production of blueberries still cannot meet the market’s demand.

Blueberries require moist but well-drained soils with a high organic matter content and low pH (4.5–5.5). For a long time, measures used for blueberry productivity promotion were mainly cultivation management, such as the use of soil amendments (peat, sawdust, etc.) to improve soil organic matter and reduce pH and/or water and fertilizer management (Holzapfel et al., 2015; Messiga et al., 2018; Yang H. et al., 2022). With the development of modern agriculture techniques, blueberry cultivation has gradually transformed from conventional to organic management. Therefore, the role of microorganisms is becoming prominent gradually. The microbes can influence plant nutrient acquisition, stress adaptation, and the control of plant disease (Berendsen et al., 2012; Cordovez et al., 2019; Trivedi et al., 2020; Zhang N. et al., 2021; Zhang Y. et al., 2021), while plants can impact soil microbial community assembly and stability (Lareen et al., 2016) as well. The rhizosphere serves as a crucial interface where interactions and mutual constraints between soil, plants, and microbes take place, alongside active zones of substance transformation (Philippot et al., 2013; Li et al., 2021; Ling et al., 2022). It is of great importance to clarify rhizosphere microbial community assembly, structure, characteristics, and its subsequent influence on plant growth, health, and productivity (Lakshmanan et al., 2014; Escudero-Martinez et al., 2022). However, little attention has been given to the interaction between microbes and blueberries. Despite studies on the beneficial inoculation of endophytic fungi or exogenous bacterial strains to blueberries (Yu et al., 2020; Guo et al., 2021), some researchers found that *Proteobacteria*, *Actinobacteria* and *Acidobacteria*, particularly *Rhizobiales* and *Pseudomonadaceae*, predominated among the rhizosphere core microbiota of blueberry cultivars and wild blueberries (Jiang et al., 2017; Morvan et al., 2020; Zhang N. et al., 2021; Zhang Y. et al., 2021). However, microbial community structures were varied due to plant age, health status and genotype (Jiang et al., 2017; Chen et al., 2019). Clarifying the blueberry rhizosphere microbial community changes under different ages and health conditions is crucial for understanding the effects of microbial community assembly and functioning on blueberry growth.

Therefore, the primary objectives of this study are to (1) characterize the rhizosphere microbial community of blueberries at various growth stages and under different health conditions, and (2) identify the core rhizobacteria and evaluate the functionality of beneficial rhizosphere bacteria in promoting blueberry growth.

## 2 Materials and methods

### 2.1 Sample collection

Soil samples were collected from the rhizosphere of blueberry cultivar O’Neal, a superea18-rly maturing and widely cultivated highbush blueberry variety (*Vaccinium corymbosum* L.). The collection site was located in the Jiangning district of Nanjing City, Jiangsu Province, China. Three fields planted with 15-year-old healthy trees (O) and 2-year-old seedlings with good (OG) and poor (OB) growth conditions were selected for rhizosphere soil sampling. The poorly growing seedlings exhibited poor growth (dwarfism) and visible foliar damage (brown patches on leaves). Rhizosphere soil samples were collected on November 18th, 2020. Each of the three plots of 10 m × 10 m was randomly selected in the O, OG, and OB fields. In each plot, five rhizosphere soil samples were randomly collected at a depth of 0–20 cm using a spade. After the loosely adhering soil was shaken off, the tightly adhering rhizosphere soil was collected into a new Falcon tube with a sterile brush. The five collected rhizosphere soil samples were then uniformly mixed into a single sample. Fresh soil (approximately 2.0 g) was transferred to a 2 mL sterile centrifuge tube, transferred to the laboratory on ice, and stored at −80°C until DNA extraction and bacterial community profiling. Simultaneously, another 2.0 g of fresh soil was stored at 4°C for bacterial isolation. Residue soil samples were air-dried, ground, and sieved for soil chemical property analysis.

### 2.2 Soil chemical properties

Soil chemical properties were examined as previously described (Lu, 2000). Briefly, soil pH and EC were determined using a 1:2 ratio of soil to deionized water with a benchtop pH meter electrode (Thermo Scientific Orion 4-Star, United States) and conductivity meter (DDS-307A, China). Soil organic matter (SOM) was determined by the Walkley-Black method. Soil total nitrogen (TN) was measured by Kjeldahl digestion and distillation. Alkaline hydrolysis nitrogen (AN) was determined by the alkaline hydrolysis diffusion method. Soil available phosphorus (AP) was extracted with 0.5 M NaHCO_3_ according to the Olsen method. Available potassium (AK) was extracted with ammonium acetate and determined using an atomic absorption spectrophotometer (Varian Spectr AA 220FS, 220Z; Varian, Palo Alto, CA).

### 2.3 DNA extraction, illumina sequencing, and processing

DNA extraction of rhizosphere soil samples was carried out using the E.Z.N.A.^®^ soil DNA Kit (Omega Biotek, Norcross, GA, United States) according to the manufacturer’s protocol. DNA quality and concentration were examined on a NanoDrop 2000 UV–vis spectrophotometer (Thermo Scientific, Wilmington, United States). Sequencing was conducted in Majorbio Bio-Pharm Technology Co., Ltd. (Shanghai, China) with primer set 338F (5′-ACTCCTACGGGAGGCAGCAG-3′) and 806R (5′-GGACTACHVGGGTWTCTAAT-3′) for V3-V4 16S rRNA gene amplification using an ABI GeneAmp^®^ 9,700 PCR thermocycler (ABI, CA, United States) (Supplementary Note S1). The purified amplicons were pooled in equimolar amounts and paired-end sequenced (2 × 300) on an Illumina MiSeq platform (Illumina, San Diego, United States) according to standard protocols. Sequence data processing was conducted through the platform of the company. Sequencing data processed according to previous studies (He et al., 2023; Zhao et al., 2024). Details of sequencing data processing can be found in Supplementary Note S2. The obtained Illumina sequences in this study had been deposited in GenBank under accession PRJNA1059765.

### 2.4 Bacteria isolation and identification

The gradient dilution method was used for blueberry rhizosphere soil bacterial strain isolation. Briefly, soil samples collected from the rhizosphere of old blueberry trees (O) were mixed evenly, and then 0.50 g was weighed and suspended in PBS buffer (pH 7.4). The soil suspension was homogenized by shaking on a Vortex-Genie 2 shaker (Scientific Industries, United States) for 30 min at 1000 rpm. After serial dilution, 100 μL of diluent was plated onto solid nutrient media (Supplementary Table S1) and incubated at 30°C. Single colonies were picked and transferred at least five times. Bacterial DNA was extracted, and the complete 16S rDNA was amplified with universal bacterial primers 27F (5′-AGAGTTTGATCMTGGCTCAG-3′) and 1492R (5′-GGYTACCTTGTTACGACTT-3′). The PCR products were analyzed and sent to Nanjing Qingke Biotechnology Co., Ltd., China, for sequencing. The obtained high-quality 16S rRNA sequences were analyzed with BLAST (NCBI)^1^ and LPSN^2^ to identify each bacterium. API ZYM and API 50 CHB/E system (Biomerieux, France) were used to analyze several typical isolates (data not shown). The phylogenetic study was carried out with MEGAX version 10.0.5. Potential nitrogen fixing and phosphorus solubilizing abilities were assessed by inoculating the strains into Ashby’s N-free medium and Pikovskaya’s agar medium according to our previously reported methods (Li et al., 2022).

### 2.5 Effect of dominant isolates on blueberry growth

The pot experiment was carried out in a greenhouse from May 10 to October 10, 2021. Blueberry seedlings of semiannual tissue culture cuttings were transplanted into a plastic pot (15 cm in diameter and 20 cm in height) with 1.0 kg air-dried soil. Four isolates, O_YPD_2, O_LB_1, O_LB_2, and O_YPD_6, were pre-inoculated in Nutrient Agar medium (NA) and incubated at 30°C for 24 h. After centrifugation, the harvested cells were washed twice with sterile water and then resuspended in 100 mL of sterile water. Finally, 25 mL of 10^6^ cfu/mL diluted living cells were added to the periphery of the blueberry root system in each pot in different directions. Sterile water treatment was used as a control. Four replicates were prepared for each treatment, and the pots were randomly placed. All treatments and other management conditions were the same. After 5 months of cultivation, the blueberry seedlings were harvested, and both shoot and root samples were washed with tap water and rinsed three times with distilled water. Plant height and shoot length were measured using a tapeline, and leaf area was measured through digital image processing technology. Biomass dry weight was determined by oven drying at 105°C for 30 min and then at 60°C to constant mass.

All data obtained were statically analyzed using the SPSS 25.0 for Windows software package. Means were compared using one-way analysis of variance (ANOVA) with Duncan’s multiple range test at the 5% level. Data are presented as the mean ± standard error.

## 3 Results

### 3.1 Soil chemical properties

Selected physicochemical properties of blueberry rhizosphere soil are listed in Table 1. The electrical conductivity (EC) values of sampled rhizosphere soil ranged from 88 to 323 μS/cm, with a maximum value of 323 μS/cm observed under the poorly growing seedlings (OB). The pH values of the rhizosphere soil samples were between 4.26 and 5.25, and the soil organic matter contents were 2.74, 4.06, and 2.43% for O, OG, and OB, respectively. The total N and available N, P, and K ranged from 1.27 to 2.01 g/kg, 94.8 to 166 mg/kg, 46 to 382 mg/kg, and 267 to 330 mg/kg, respectively.

**Table 1:** Selected physical and chemical properties of blueberry rhizosphere soil.

Sample	pH	EC	SOM	TN	AN	AP	AK
		(μS/cm)	(g/kg)	(g/kg)	(mg/kg)		
O	4.26 ± 0. 1 b	122 ± 11 b	27.4 ± 1.3 b	1.89 ± 0.2 b	166 ± 12.1 a	382 ± 23 a	286 ± 15 b
OG	5.22 ± 0. 2 a	88.0 ± 5.6 c	40.6 ± 2.0 a	2.01 ± 0.2 a	155 ± 10.5 a	89 ± 9.6 b	330 ± 25 a
OB	5.35 ± 0. 2 a	323 ± 40 a	25.3 ± 1.1 b	1.27 ± 0.1 c	94.8 ± 8.1 b	46 ± 4.1 c	267 ± 6.9 b

Healthy old tree (O), young seedlings (OG), and poorly growing seedlings (OB) of O’Neal blueberry. Soil electrical conductivity (EC), soil organic matter (SOM), soil total nitrogen (TN), available nitrogen (AN), available phosphorus (AP), available potassium (AK). Different lowercase letters in each column indicate significant differences at *p* < 0.05 (Duncan).

### 3.2 Microbial rhizosphere diversity

The 16S rRNA gene sequencing results were employed to investigate bacterial diversity and community composition changes in blueberry rhizosphere soil. A total of 672,403 high-efficiency and high-quality optimized sequences with an average length of 414 bp were detected. Operational taxonomic units (OTUs) were defined at 97% similarity, resulting in a total of 4,102 OTUs gathered after weighting according to the minimum number of sample sequences. The estimators of the alpha diversity index are shown in Table 2. The coverage index ranged from 0.982 to 0.990, indicating sufficient sequencing depth. One-way ANOVA results revealed that the Sobs, Chao1, and Shannon indexes were significantly different among O, OG, and OB (*p* < 0.001), following the order OB > OG > O. However, the Berger–Parker and Simpson-Stokes indexes for OB were smaller than those for O and OG. These results indicated that poorly growing seedlings (OB) possessed higher microbial diversity and richness but lower evenness and dominance diversity.

**Table 2:** Estimators of the alpha diversity index.

Sample	97% similarity					
	Sobs	Chao	Simpson even	Shannon	Berger-Parker	Coverage
O	1,145 ± 21 c	1,378 ± 29 c	0.070 ± 0.007 b	5.33 ± 0.08 c	0.050 ± 0.006 a	0.990 ± 0.001 a
OG	1,396 ± 22 b	1731 ± 30 b	0.071 ± 0.003 b	5.61 ± 0.04 b	0.049 ± 0.001 a	0.987 ± 0.000 b
OB	1917 ± 84 a	2,360 ± 135 a	0.126 ± 0.010 a	6.34 ± 0.09 a	0.025 ± 0.004 b	0.982 ± 0.001 c

Healthy old tree (O), young seedlings (OG), and poorly growing seedlings (OB) of O’Neal blueberry. Different lowercase letters in each column indicate significant differences at *p* < 0.05 (Duncan).

**Figure 1:**
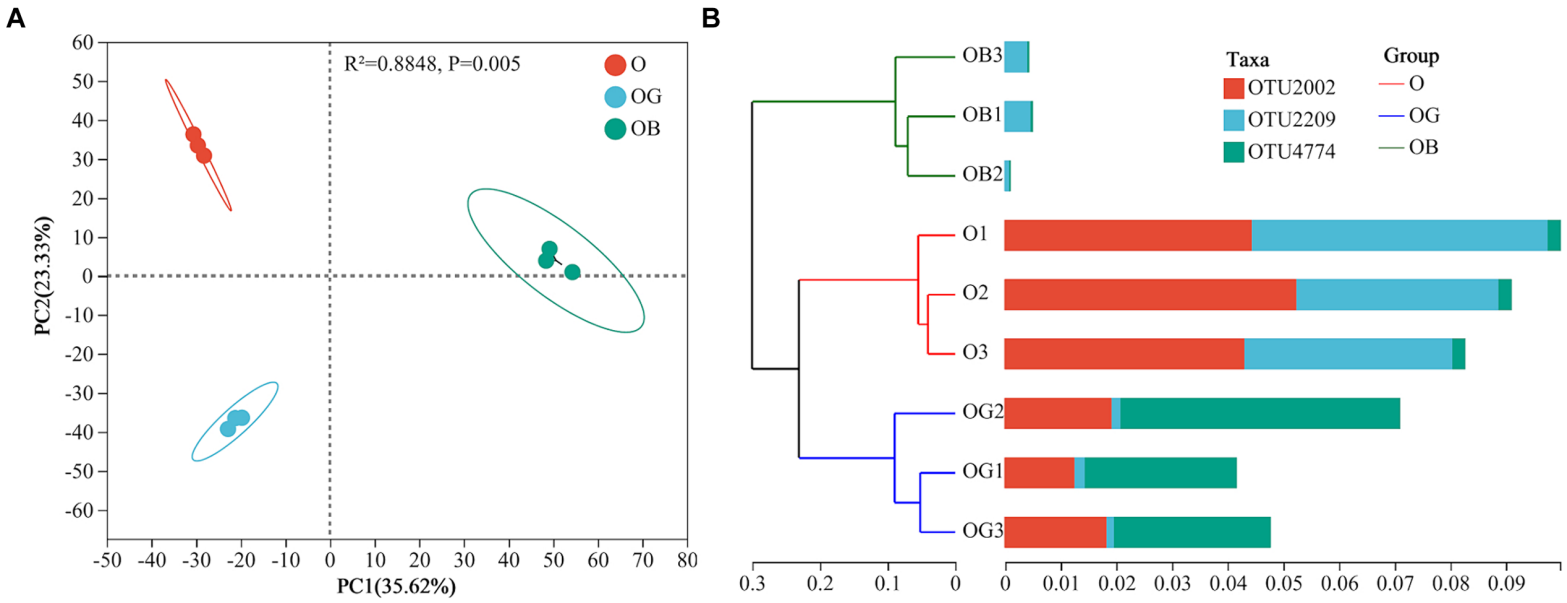
Principal component analysis **(A)** and hierarchical cluster tree **(B)** of the blueberry rhizosphere soil samples. Healthy old tree (O), young seedlings (OG), and poorly growing seedlings (OB) of O’Neal blueberry.

Visualized principal component analysis (PCA) based on Bray–Curtis distances presented in Figure 1A was employed to determine the bacterial community composition among samples (β diversity). The first principal coordinate (PC 1) revealed that the rhizosphere-associated bacterial microbiotas of healthy O and OG plants were more homogeneous than those of the communities retrieved from poorly growing OB (Figure 1A), indicating that healthy plants possessed more similar rhizosphere microbial communities. Even so, differences in rhizobacterial communities between O and OG were still existed as revealed by PC2. The hierarchical cluster tree presented in Figure 1B clearly shows that healthy plants possessed similar rhizosphere microbial communities and higher amounts of dominant taxa than poorly growing OB plants.

### 3.3 Microbial rhizosphere community composition

**Figure 2:**
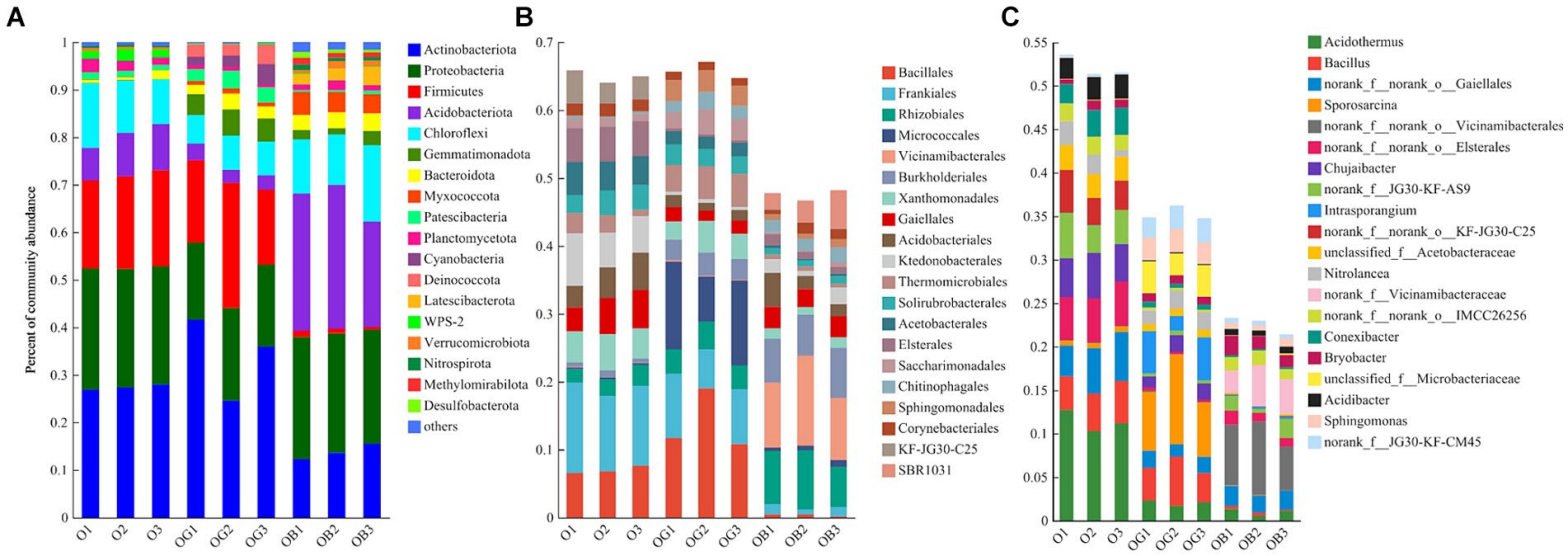
Community relative abundance of blueberry rhizosphere samples at the phylum level **(A)** and the top 20 orders **(B)** and genera **(C)**. Healthy old tree (O), young seedlings (OG), and poorly growing seedlings (OB) of O’Neal blueberry.

The dominant bacterial phyla, order, and genera were chosen to determine changes in the rhizosphere soil microbial taxonomic composition among O, OG, and OB. The *Actinobacteria*, *Proteobacteria*, *Firmicutes*, *Chloroflexi,* and *Acidobacteria* were the top five phyla, accounting for 91.7, 81.3, and 79.5% of the relative abundances of O, OG, and OB, respectively (Figure 2A).

Although the blueberry rhizosphere harbored an overall similar bacterial community, we observed some differences in the healthy (O and OG) and poorly (OB) growing plants. The healthy plants were dominated by higher levels of *Firmicutes*, whereas the OB group had a higher abundance of *Acidobacteria*. In addition to the prevailing orders *Rhizobiales* and *Gaiellales*, *Bacillales* and *Frankiales* were the most abundant orders in the rhizosphere of healthy plants (O and OG), whereas *Vicinamibacterale* had the highest relative abundance in the rhizosphere of poorly growing blueberries (OB). At the genus level, the genera *Acidothermus* (11.4%), *g_norank_f_norank_o_Elsterales* (5.0%), *norank_f_norank_o_Gaiellales* (4.8%), *Chujaibacter* (4.7%), and *Bacillus* (4.3%) predominated the O group rhizosphere; *Sporosarcina* (7.7%), *Bacillus* (4.3%), *Intrasporangium* (3.8%), and *unclassified_f_Microbacteriaceae* (3.3%) predominated the OG group rhizosphere; and *norank_f_norank_o_Vicinamibacterales* (6.8%) and *norank_f_Vicinamibacterales* (3.7%) predominated the OB group rhizosphere.

### 3.4 Core rhizobacteria, biomarkers, and phenotypic function prediction

Venn diagram analysis was further introduced to identify the common core and unique microbiomes among multiple bacterial communities sampled from healthy (O and OG) and poorly (OB) growing blueberry rhizospheres. A total of 494 bacterial OTUs were present in all blueberry rhizosphere samples, and the top 18 OTUs (>1%) belonged to four predominant phyla: *Actinobacteria*, *Proteobacteria*, *Firmicutes*, and *Chloroflexi* (Supplementary Figure S2; Supplementary Table S3). Among them, OTU2411 (norank_o_*Gaielales*) and OTU2907 (*Bradyrhizobium*) had high relative abundance in both healthy and poor-growing blueberry rhizospheres. In addition, OTU2002 (*Chujaibacter*), OTU1765 (*Acidothermus*), and OTU2112 (*Bacillus*) were enriched only in the healthy blueberry rhizosphere. Furthermore, 1,433 unique OTUs were observed in the poorly growing blueberry rhizosphere, and the predominant bacteria (>1%) mainly belonged to the phylum *Acidobacteria* (OTU3271, 3,224, 3,678, and 2,941), followed by *Latescibacterota* (OTU4008) (Supplementary Figure S2; Supplementary Table S4).

**Figure 3:**
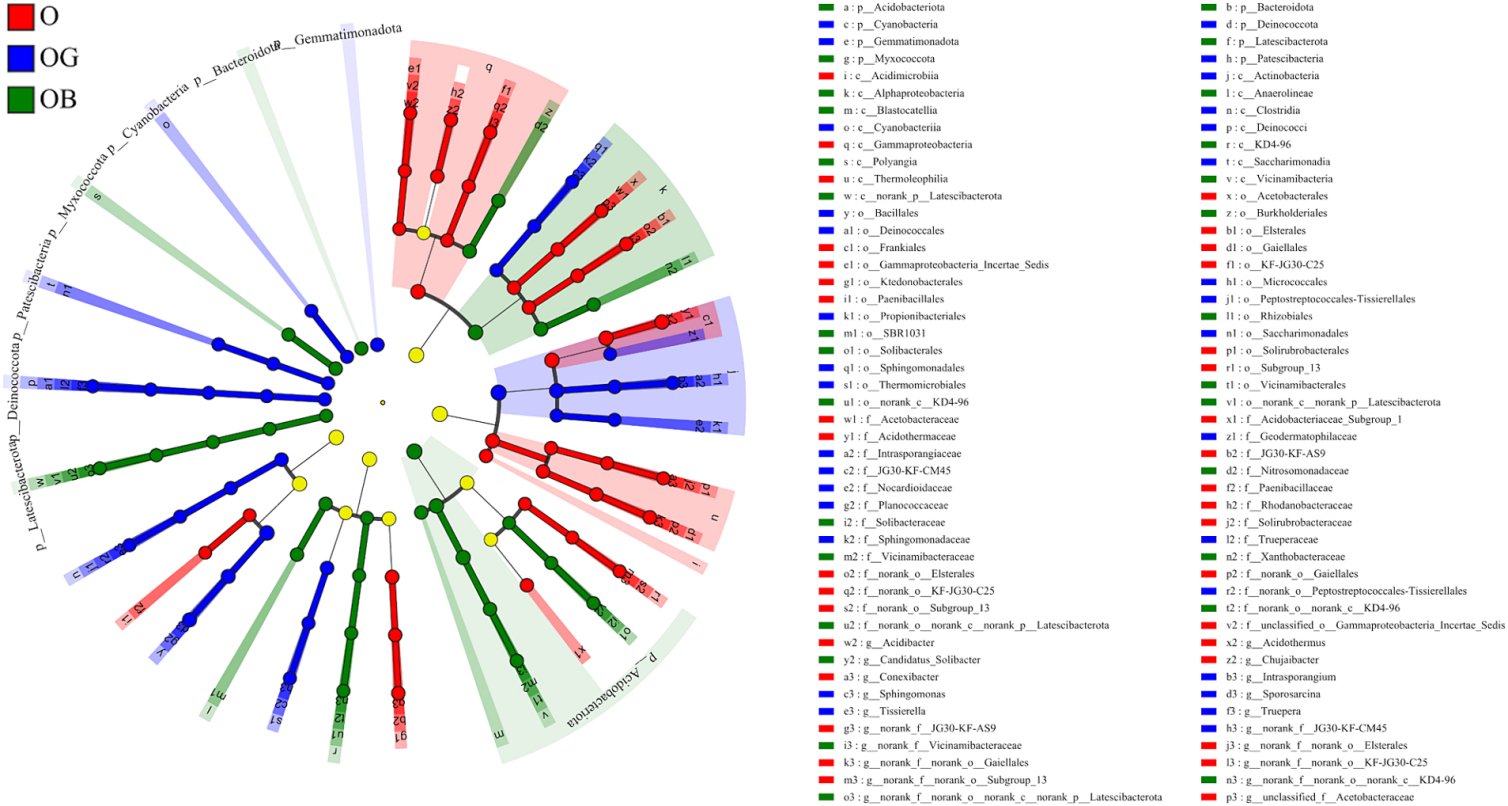
Cladogram displaying the taxonomic hierarchy distribution of marker species significantly enriched in O, OG, and OB (*p* ≤ 0.05, LDA ≥ 4). Healthy old tree (O), young seedlings (OG), and poorly growing seedlings (OB) of O’Neal blueberry.

The linear discriminant analysis (LDA) effect size was employed to determine biomarkers enriched in different samples. A total of 94 bacterial clads presented statistically significant differences with a threshold of 4 (Figure 3). Among them, 35, 31, and 28 clads were identified as biomarkers in groups O, OG and OB, respectively. *Frankiales*, *Acidothermaceae,* and *Acidothermus* were the three most significant biomarkers in the O group. The three most significant biomarkers in the OG group were *Actinobacteria*, *Bacillales,* and *Micrococcales*, and those in the OB group were *Acidobacteriota*, *Vicinamibacterales,* and *Vicinamibacteria*.

**Figure 4:**
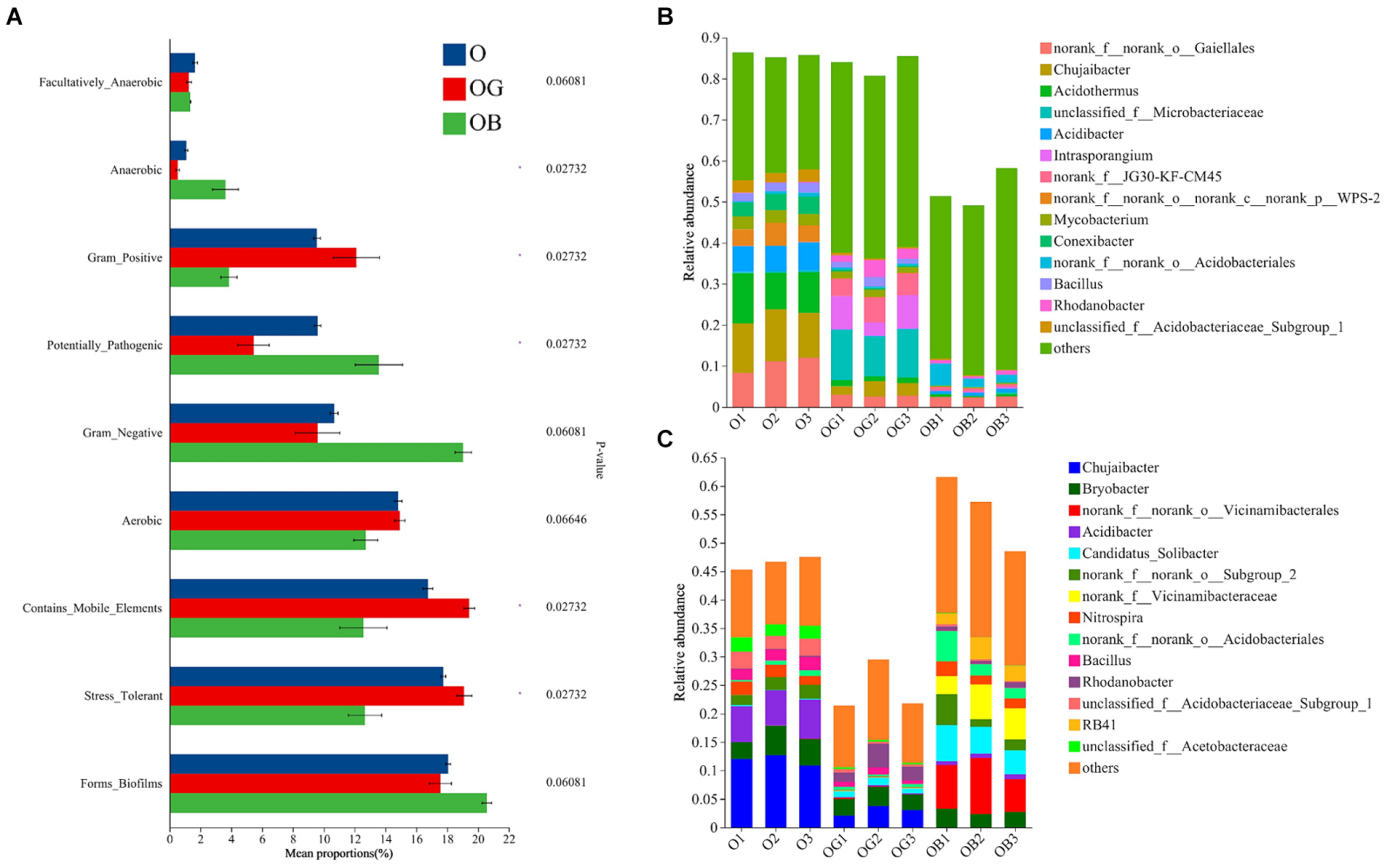
Rhizobacterial community phenotypic differences among O, OG and OB predicted by BugBase **(A)** and species contributions to stress tolerance **(B)** and potential pathogenicity **(C)**. Healthy old tree (O), young seedlings (OG), and poorly growing seedlings (OB) of O’Neal blueberry.

Furthermore, we used BugBase to predict the phenotypic function of rhizobacteria to explore the keystone bacteria that affected the blueberry rhizosphere microbial community function. Compared with healthy blueberry rhizobacteria, the relative abundances of phenotypes of stress-tolerant, contains_mobile_elements and gram_positive significantly decreased and those of potentially pathogenic microbes dramatically increased in the rhizobacteria of poorly growing blueberry (Figure 4A). Specifically, the predicted phenotype of stress tolerance in OB rhizobacteria decreased by 36.9 and 37.1% relative to the groups of O and OG, respectively. Furthermore, *norank_f_norank_o_Gaiellales* had a high relative abundance and contributed to the phenotype of the stress tolerance in all blueberry rhizobacterial samples, indicating its important role in blueberry rhizosphere stress resistance (Figure 4B). In addition, the genera *Chujaibacter*, *Acidothermus*, *Mycobacterium*, and *Bacillus* also contributed to the phenotype of stress tolerance in the healthy blueberry rhizosphere (>1%), but they had almost no contribution in the OB rhizosphere (<1‰). Meanwhile, the predicted phenotypes of potentially pathogenic in OB rhizobacteria were 1.25 and 2.36 times those of O and OG rhizobacteria, respectively. Among them, *norank_f_norank_o_Vicinamibacterale* and *norank_f_Vicinamibacterale* contributed more than 10% of the potentially pathogenic bacteria in OB (Figure 4C).

### 3.5 Rhizosphere microbial correlation with environmental factors

**Figure 5:**
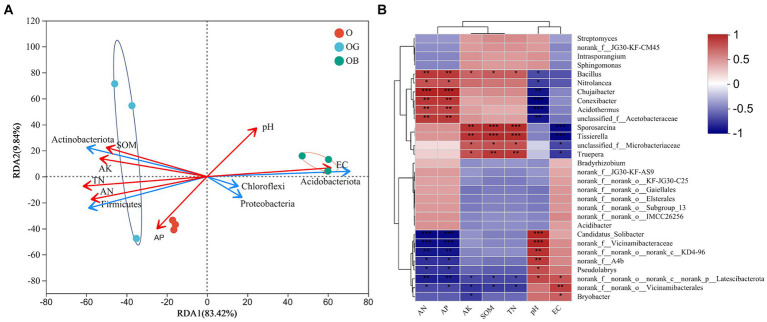
Correlation analysis between the microbial community structure and soil chemical properties. **(A)** RDA (redundancy analysis) at the phylum level. **(B)** Spearman correlation at the genus level. Healthy old tree (O), young seedlings (OG), and poorly growing seedlings (OB) of O’Neal blueberry. Soil electrical conductivity (EC), soil organic matter (SOM), soil total nitrogen (TN), available nitrogen (AN), available phosphorus (AP), available potassium (AK). Significance level: *p ≤ 0.05; **p ≤ 0.01; ***p ≤ 0.001.

Redundancy analysis (Figure 5A) showed that among the five most abundant phyla, *Firmicutes* and *Actinobacteria* had similar correlations with environmental factors, namely, they were positively correlated with AN and AP (*p* < 0.01), AK, TN, and SOM (*p* < 0.05) and negatively correlated with pH and EC (*p* < 0.05). By contrast, the relative abundances of *Proteobacteria*, *Chloroflexi,* and *Acidobacteria* were negatively correlated with available K, SOM, and total N (*p* < 0.05) but positively correlated with EC (*p* < 0.05). At the genus level, the Spearman correlation heatmap showed that *Bacillus* strongly interacted with environmental factors, the correlation of which with environmental factors was the same as that of *Firmicutes*. In addition, the genera *Nitrolancea*, *Chujaibacter*, *Conexibacter*, *Acidothermus*, *unclassified_f_Acetobacteraceae*, *Sporosarcina*, *Tissierella*, *unclassified_f_Microbacteriaceae* and *Truepera* showed similar correlations with environmental factors as *Bacillus*. Notably, the correlations of the genera *Candidatus_Solibacter*, *norank_f_o_norank_c_KD4-96*, *norank_f_A4b*, *Pseudolabrys*, *Bryobacter*, *norank_f_Vicinamibacteraceae*, and especially *norank_f_norank_o_Vicinamibacterales* and *norank_f_norank_o_nornak_c_norank_p_Latescibacterota* with environmental factors were opposite to those of *Bacillus*.

Two-way correlation network analysis was employed to further illustrate the relationship between microbiomes and environmental factors. The genera *Bacillus*, *norank_f_norank_o_Vicinamibacterales* and *norank_f_norank_o_nornak_c_norank_.*

**Figure 6:**
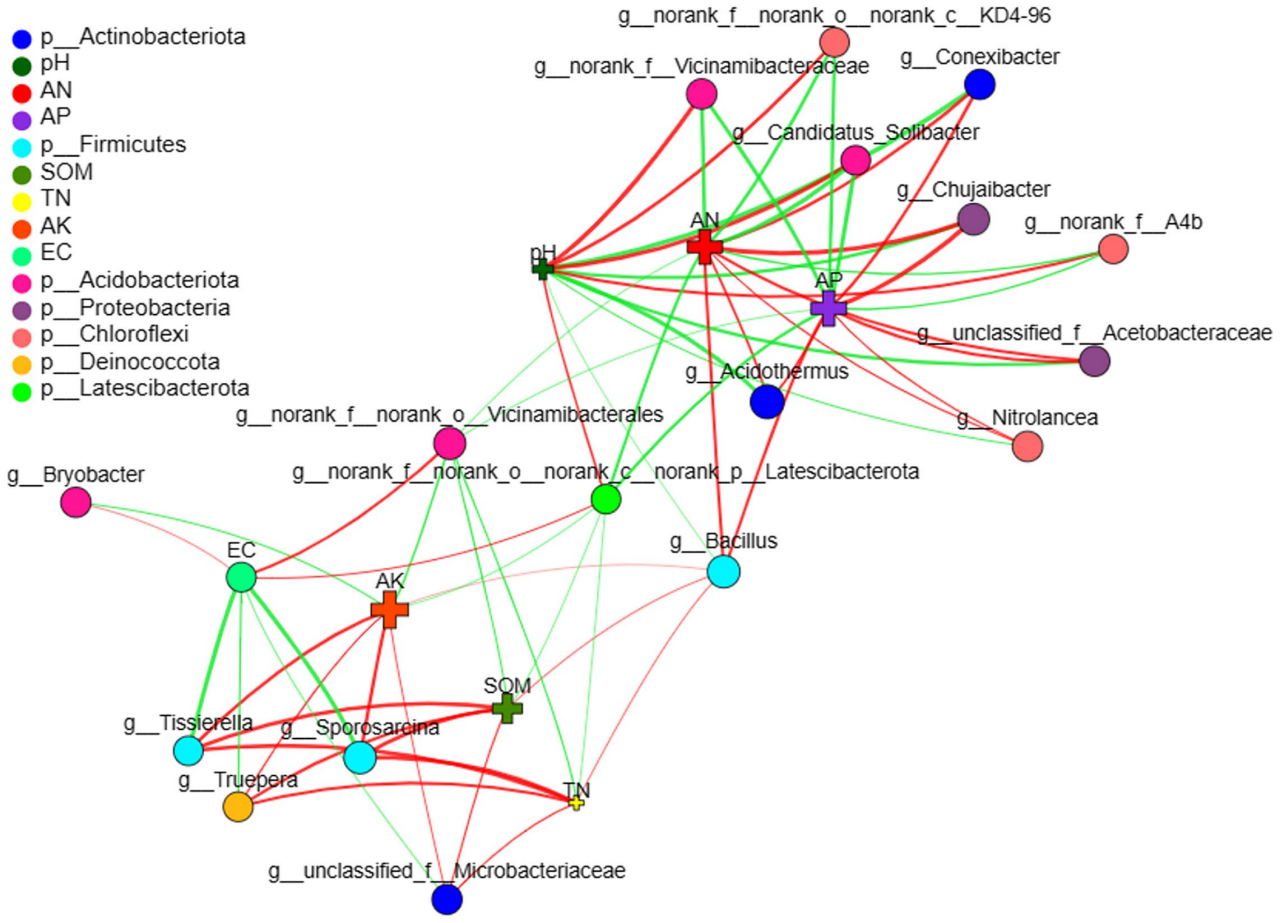
Two-way correlation network of blueberry rhizobacterial and environmental factors. Soil electrical conductivity (EC), soil organic matter (SOM), soil total nitrogen (TN), available nitrogen (AN), available phosphorus (AP), available potassium (AK).

*p_Latescibacterota* were the most important nodes of blueberry rhizobacteria, which connected the microbial networks and environmental factors (Figure 6). In addition, AN, AP, and pH were the most important nodes of environmental factors that affected the rhizosphere microbial community. *Bacillus* showed a positive correlation with the environmental factors (nutrients) that benefit blueberry growth, such as AN, AP, AK, and SOM, whereas *norank_f_norank_o_Vicinamibacterales* and *norank_f_norank_o_nornak_c_norank_p_Latescibacterota* exhibited a negative correlation with nutrients but a positive correlation with EC.

### 3.6 Microbial isolation and plant growth promotion

**Figure 7:**
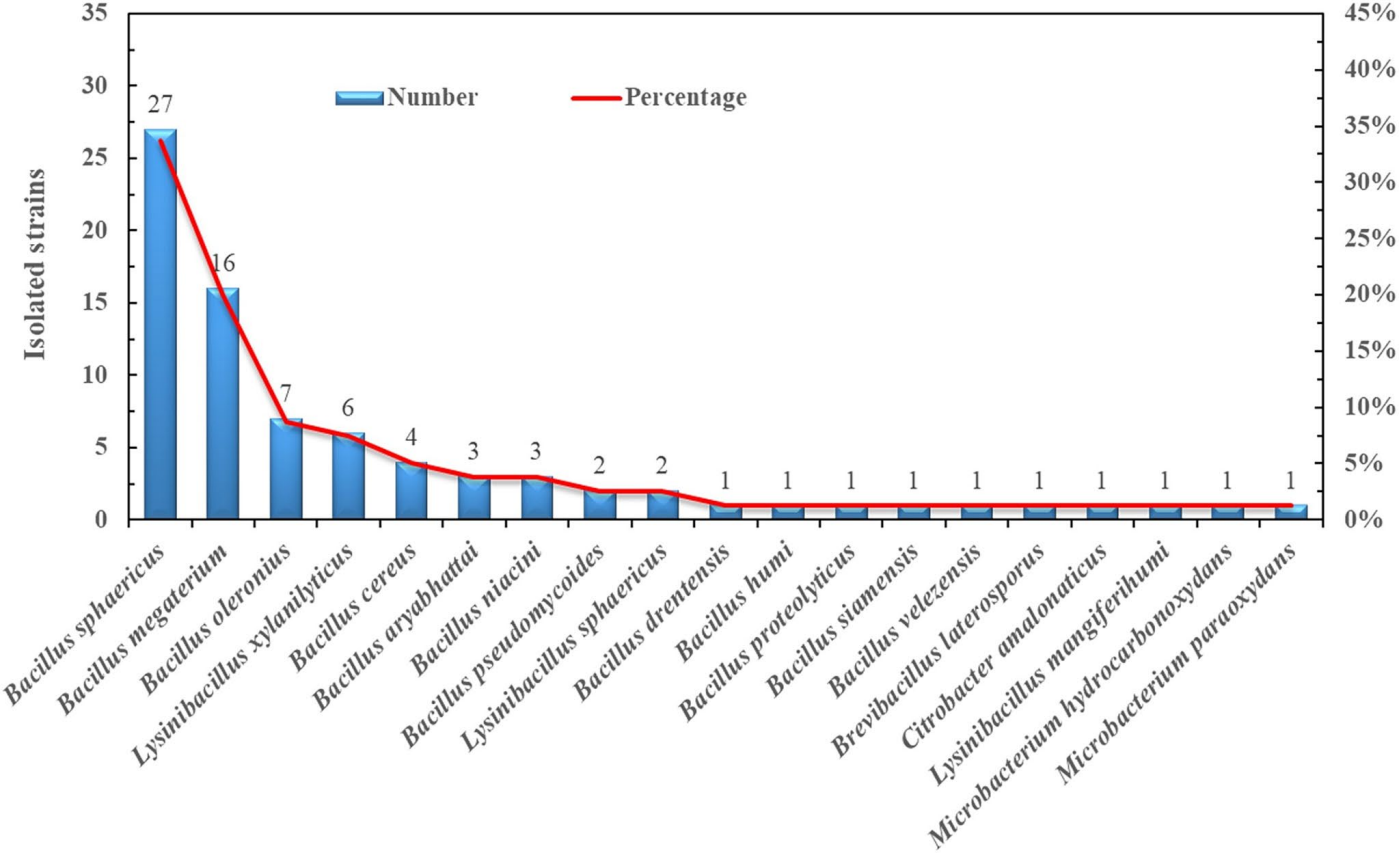
Number and percentage of the isolated strains from the rhizosphere of old blueberry tree (O).

A total of 80 bacteria were isolated and identified from the rhizosphere soil of the old trees (O) (Figure 7; Supplementary Table S5). The predominant genus was *Bacillus*, which accounted for 83.8% of the isolates. This result was in accordance with the Illumina sequencing data showing that *Bacillus* was one of the key genera in the healthy blueberry rhizosphere (Figure 2). Specifically, 27 *Bacillus sphaericus* and 16 *Bacillus megaterium* strains were obtained, accounting for 34 and 20% of the isolates, respectively. Other abundant species were *Bacillus Polonius* (9%), *Lysinibacillus xylaniyticus* (8%), and *Bacillus cereus* (5%). The growth of the isolates on nitrogen-free Ashby medium and Pikovskaya’s agar medium was recorded (Supplementary Table S5). Ten out of 16 *Bacillus megaterium* strains grew well in the Ashby medium, whereas 13 isolates were able to induce clear circle zones on phosphorus-containing agar medium, indicating high potential nitrogen-fixing and phosphorus-solubilizing capacity of *Bacillus megaterium* isolates such as strain O_LB_2 (Supplementary Table S5). *Bacillus cereus* and *Bacillus oleronius* strains also had high potential for nitrogen-fixing and phosphorus-solubilizing abilities, similar to *B. megaterium* stain O_LB_2. Conversely, *Bacillus sphaericus* strains barely grew on Ashby and Pikovskaya’s agar medium.

**Figure 8:**
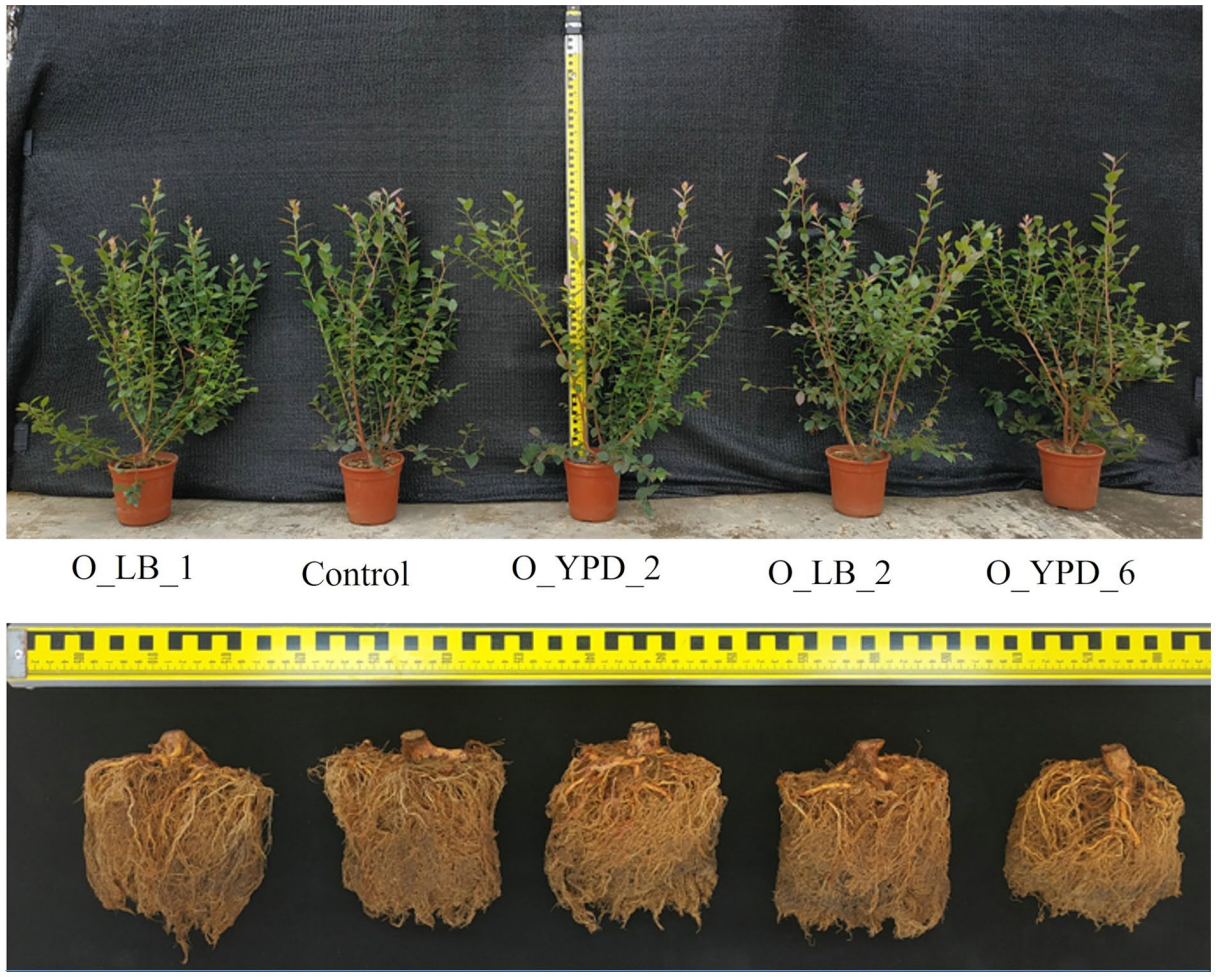
Effects of bacterial strains on the growth of the blueberry trees and the roots after 5 months of cultivation.

Blueberry growth was promoted by the inoculation of various *Bacillus* strains (Figure 8; Table 3). The plant growth-promoting effect of the selected strains followed the order O_LB_2 > O_YPD_6 > O_LB_1 > O_YPD_2 (Table 3). Specifically, inoculation with stain O_LB_2 improved plant height, shoot length, leaf area, and total dry biomass by 32.5, 14.3, 75.6, and 36.2%, respectively. Leaf area and dry weight of root and shoot were significantly enhanced by inoculation with strain O_YPD_6 by 33.3, 33.8, and 19.1%, respectively. Inoculation with strain O_LB_1 significantly enhanced the leaf area and root dry weight by 24.2 and 25.0%, respectively. However, the growth of blueberries was not affected by inoculation with strain O_YPD_2, although the average values were higher than those of the control.

**Table 3:** Selected physiological parameters of blueberries with and without root inoculation treatment.

Treatment	Plant Height (cm)	Shoot length (cm)	Leaf area (cm^2^)	Dry weight(g)			
				Root	Shoot	Leaf	Total
Control	62.8 ± 2.8 b	1,058 ± 38 b	2,754 ± 449 c	8.0 ± 1.0 b	37.2 ± 1.2 c	30.3 ± 1.1 c	75.4 ± 2.0 c
O_YPD_2	63.1 ± 2.5 b	1,084 ± 29 b	2,973 ± 350 c	8.3 ± 0.6 b	38.0 ± 1.3 c	30.9 ± 0.7 c	77.2 ± 0.9 c
O_YPD_6	65.2 ± 1.3 b	1,157 ± 63 b	3,670 ± 139 b	10.7 ± 0.5 a	44.3 ± 1.1 b	35.8 ± 1.6 b	90.7 ± 2.6 b
O_LB_1	63.3 ± 3.7 b	1,079 ± 32 b	3,420 ± 496 b	10.0 ± 0.5 a	37.8 ± 0.6 c	31.1 ± 0.8 c	78.9 ± 1.3 c
O_LB_2	83.2 ± 3.3 a	1,209 ± 91 a	4,835 ± 595 a	11.7 ± 0.7 a	47.6 ± 1.3 a	43.4 ± 1.7 a	102.7 ± 3.8 a

Different letters in the same column indicate significant differences (*p* < 0.05) among the five treatments. Means of four replicates ± standard error. Control represents no bacterial inoculation; O_YPD_2, O_YPD_6, O_LB_1, and O_LB_2 represent the inoculation of the isolated *Bacillus sphaericus*, *Bacillus oleronius*, *Bacillus cereus*, and *Bacillus megaterium*, respectively.

## 4 Discussion

### 4.1 Effect of soil chemical properties on blueberry growth

Blueberries thrive in well-drained soils with high organic matter content and low pH and electrical conductivity (Spiers and Braswell, 1992; Drummond et al., 2009; Caspersen et al., 2016). In the present study, the rhizosphere soil samples had pH values ranging from 4.26 to 5.35 and EC values between 122 and 323 μS/cm (Table 1), indicating that the soils were suitable for blueberry cultivation (Chen et al., 2019). An organic matter content of at least 30 g/kg is recommended for optimal blueberry growth. Recently, Dong et al. (2016) proposed a five-grade classification system for evaluating the organic matter content of blueberry orchard soils in China (Supplementary Table S2; Dong et al., 2016). According to this classification, the soil organic matter contents in the study area ranged from 24.3 to 40.6 g/kg (Table 2), classified as grades 2 (OG) and 3 (O and OB), respectively (Supplementary Table S2). The SOM content of the OB group was slightly lower than that of the OG group but approximately equal to that of the O group. Therefore, SOM content alone should not be considered the key factor affecting the growth of the OB group.

However, the rhizosphere soil total nitrogen, available nitrogen, phosphorus, and potassium of the OB group were significantly lower than those of the healthy (O and OG) blueberry plants (Table 1; Supplementary Table S2). Specifically, the soil total and available nitrogen of the OB group decreased to grade 3, and the available phosphorus decreased to 46 mg/kg, although it was still at the grade 1 level. Blueberries are considered an oligotrophic plant, meaning they have relatively low nutritional requirements. Even though the soil nutrient levels of the OB group were relatively lower than the healthy plants, they were still at a medium to high level, which should be sufficient to support blueberry growth.

### 4.2 Rhizobacterial diversity and community composition

Rhizosphere microbial communities significantly influence plant growth, health, and productivity (Lakshmanan et al., 2014). In this study, we found that the rhizosphere bacterial community diversity decreased in the order OB > > OG > O (Table 2). This contrasts with the findings by Chen et al. (2019), who reported higher microbial diversity in the rhizosphere of healthy blueberry plants compared to poor-performing plants (Chen et al., 2019). Host plants regulate the composition of their rhizosphere microbiome by providing selective environments and manipulating root exudates (Chaparro et al., 2014; Lareen et al., 2016). Factors like plant age, nutritional requirements, environmental conditions, and abiotic stresses influence the composition of root exudates, thereby shaping the rhizosphere microbial community (Zhalnina et al., 2018; Hou et al., 2019).

We speculate that after 15 years of cultivation, the unique and stable root exudate profile and microbial substrate preferences of mature plants (O group) have driven the recruitment of specific taxa, leading to a beneficial root microbiota community. In contrast, the rhizosphere microbiome of newly planted seedlings (OG group) may be more influenced by the indigenous soil microorganisms during initial growth stages. Over time, these native microorganisms are selected for and enriched, ultimately resembling the microbial community of mature plants (Nuccio et al., 2020). This may explain the higher diversity observed in the newly planted seedlings compared to mature plants. Consistent with this, a study by Dong et al. (2018) found that rhizosphere microbial diversity and richness of blueberries decreased with cultivation duration (9 years <6 years <5 years) (Dong et al., 2018). Environmental factors and abiotic stresses further shape the microbial community (Zhalnina et al., 2018). PCA (Figure 1A) and hierarchical clustering (Figure 1B) results indicated that microbial community composition was primarily influenced by blueberry health and age, with well-growing seedlings (OG) being more similar to old trees (O), while poorly growing plants (OB) exhibited a distinct microbial community.

The dominant phyla in the O’Neal blueberry rhizosphere included *Actinobacteria*, *Proteobacteria*, *Firmicutes*, *Chloroflexi*, and *Acidobacteria* (Figure 3A). Previous studies reported *Proteobacteria*, *Actinobacteria*, *Acidobacteria*, and *Chloroflexi* as core rhizosphere microbiotas of highbush and rabbiteye blueberry (Yurgel et al., 2017; Chen et al., 2019; Zhang N. et al., 2021; Zhang Y. et al., 2021). However, *Firmicutes* were rarely predominant, except in a study by Guo et al. (2021), which noted their dominance after mycorrhizal fungi (*Penicillium pinophilum*) inoculation (Guo et al., 2021). We also found *Rhizobiales* (*Bradyrhizobium*) and *Gaiellales* (norank_o_Gaiellales) common in both healthy and poorly growing rhizospheres (Figure 3B; Supplementary Figure S1). Morvan et al. (2020) indicated that *Bradyrhizobium* sp. could play a key role in nitrogen cycling, enhancing wild blueberry growth. *Gaiellales* have been found to significantly enhance stress tolerance (Figure 6B; Morvan et al., 2020), boost the abundance and activity of beneficial soil bacteria (Ding et al., 2021), and promote the accumulation of polysaccharides, thereby improving plant growth and health (Liu et al., 2022).

Differences in rhizobacteria between healthy (O and OG) and poorly growing (OB) plants were evident. Samples from O and OG had higher levels of *Actinobacteria* and *Firmicutes* with lower *Acidobacteriota* abundance, while OB showed the opposite (Figure 2A; Supplementary Figure S2). Chen et al. (2019) also found that *Actinobacteria* were more abundant in the rhizosphere soil of healthy plants than in poorly growing ones. *Actinobacteria* is an important phylum because some species are useful for plant growth promotion and combating abiotic and biotic stresses through nitrogen fixation, P and K solubilization, phytohormone production, improving the decomposition of organic matter, and secreting antimicrobial compounds to suppress soil-borne plant pathogens (Mitra et al., 2022). *Firmicutes*, including genera such as *Bacillus*, *Planococcus*, *Sporosarcina*, *Terribacillus*, and *Lysinibacillus*, are well-documented for their roles as plant growth-promoting organisms and biocontrol agents, and they have been utilized in biofertilizers (Hashmi et al., 2020). We observed positive correlations between the abundances of *Actinobacteria* and *Firmicutes* and soil parameters such as organic matter, available nitrogen, total nitrogen, phosphorus, and potassium (Figure 5A), which may contribute to better soil quality, nutrient cycling, and availability for plants thereby potentially enhancing blueberry growth and health.

In contrast, *Acidobacteriota* are typically enriched in nutrient-poor soils and are important in the degradation of polysaccharides (Beckers et al., 2017). Previous studies on wild blueberry rhizosphere microbiota have reported that *Acidobacteriota* was the most abundant phylum (Yurgel et al., 2017; Morvan et al., 2020). In the present study, *Acidobacteriota* was the most abundant phylum in the rhizosphere of poorly growing blueberry plants (OB), predominated by the order *Vicinamibacterales* (Figure 3B). Conversely, the relative abundance of *Acidobacteriota* significantly decreased in healthy plants (Supplementary Figure S2), and the predominant class was *Acidobacteriae*, consisting with previous findings (Morvan et al., 2020).

### 4.3 Keystone rhizosphere microorganisms

Keystone bacteria play crucial roles in shaping rhizosphere microbial communities, impacting soil properties and plant productivity (Banerjee et al., 2018; Yang et al., 2021). In our study, *Gaiellales* and *Bradyrhizobium* were identified as core microbiomes in the O’Neal blueberry rhizosphere (Supplementary Table S3; Supplementary Figure S3), consistent with previous reports (Jiang et al., 2017; Morvan et al., 2020). *Bacillus*, *Vicinamibacterales*-related, and *Latescibacterota*-related genera emerged as key nodes in the rhizobacterial network (Figure 6).

*Bacillus* showed significant correlations with SOM, total and available N, and available P, suggesting its role in enhancing nutrient and energy cycling (Miljakovic et al., 2020). *Bacillus*, a well-studied PGPR, positively affects growth and biocontrol in various crops (Aloo et al., 2019; Lu et al., 2022; Feng et al., 2024). However, its role in blueberry growth was previously unreported, except for controlling gray mold (Lu et al., 2021). Here, *Bacillus* was a keystone in healthy blueberry rhizospheres, constituting over 80% of isolated bacteria (Supplementary Table S5) but was deficient in poorly growing plants (Figure 2C). Inoculating seedlings with *Bacillus*, especially *Bacillus megaterium*, significantly enhanced growth (Table 3). This is the first report indicating *Firmicutes* (*Bacillus*) as a keystone in the O’Neal blueberry rhizosphere.

*Vicinamibacterales*-related (6.8%) and *Latescibacterota*-related (2.7%) genera were abundant in poorly growing blueberry rhizospheres, identified as unique biomarkers (Supplementary Table S4; Figure 3). Chen et al. (2019) suggested that exclusive microorganisms might be pathogenic or impair soil nutrient cycling, affecting plant growth negatively (Chen et al., 2019). Our study found that *Vicinamibacterales* and *Latescibacterota* abundances correlated positively with EC and soil pH, but negatively with SOM and nutrients (Figure 5B). Blueberries prefer soils with high SOM but low pH and EC, indicating a habitat mismatch in poorly growing plants. Historically, fungi and nematodes, not bacteria, were considered the main pathogens for blueberries (Song et al., 2021). We observed a higher abundance of potentially pathogenic bacteria in poorly growing blueberries, with *Vicinamibacterales* contributing over 10% (Figures 4A,C). This suggests that *Latescibacterota* and *Acidobacteriota* (order *Vicinamibacterales*) significantly influence rhizobacterial community composition and function in poorly growing blueberries, with some *Vicinamibacterales* strains potentially acting as pathogenic bacteria.

## 5 Conclusion

The rhizosphere of O’Neal blueberries is predominantly inhabited by bacteria from the phyla *Actinobacteria*, *Proteobacteria*, *Firmicutes*, *Chloroflexi*, and *Acidobacteria*. However, the diversity and community structure of these bacteria vary depending on the age and growth conditions of the blueberries. Healthy blueberry plants (O and OG) demonstrated an increased presence of *Actinobacteria* and *Firmicutes*. Notably, the *Firmicutes* phylum, which has not been commonly found to be predominant in previous studies, was particularly enriched in the rhizosphere of healthy blueberries, with *Bacillus* emerging as a crucial genus. Furthermore, inoculation with *Bacillus megaterium*, isolated from this environment, significantly boosted blueberry growth, underscoring *Bacillus* as a vital rhizobacterium with considerable agricultural potential. In contrast, blueberries exhibiting poor growth (OB) were associated with higher levels of *Acidobacteria*, particularly genera related to *Vicinamibacterales* and *Latescibacterota*, identified as keystone taxa. These taxa had a substantial impact on the microbial community structure of OB and were predicted to be potential pathogenic bacteria. This study highlights the influence of keystone bacteria on shaping and functioning the blueberry rhizosphere microbial community. *Bacillus* stands out as one of the core rhizobacteria with promising future applications in blueberry cultivation.

## Data Availability

The datasets presented in this study can be found in online repositories. The names of the repository/repositories and accession number(s) can be found at: https://www.ncbi.nlm.nih.gov/, PRJNA1059765.

## Footnotes

^1^ http://blast.ncbi.nlm.nih.gov

^2^ https://lpsn.dsmz.de/
