# Supplementary material for: Rhizobacterial diversity, community composition, and the influence of keystone taxa on O’Neal blueberry (Vaccinium corymbosum)
Source: Front Microbiol. 2024 Sep 13;15:1460067. doi: 10.3389/fmicb.2024.1460067 (PMC11427291; doi:10.3389/fmicb.2024.1460067)
Supplement: Supplementary file 1 [file Data_Sheet_1.DOCX]

Supplementary Material

# Supplementary Note 1: PCR amplification

The PCR amplification of 16S rRNA gene was performed as follows: initial denaturation at 95 ℃ for 3 min, followed by 27 cycles of denaturing at 95 ℃ for 30 s, annealing at 55 ℃ for 30 s and extension at 72 ℃for 45 s, and single extension at 72 ℃ for 10 min, and end at 4 ℃. The PCR mixtures contain 5 × *TransStart* FastPfu buffer 4 μL, 2.5 mM dNTPs 2 μL, forward primer (5 μM) 0.8 μL, reverse primer (5 μM) 0.8 μL, *TransStart* FastPfu DNA Polymerase 0.4 μL, template DNA 10 ng, and finally ddH_2_O up to 20 μL. PCR reactions were performed in triplicate. The PCR product was extracted from 2% agarose gel and purified using the AxyPrep DNA Gel Extraction Kit (Axygen Biosciences, Union City, CA, USA) according to manufacturer’s instructions and quantified using Quantus™ Fluorometer (Promega, USA).

# Supplementary Note 2: Processing of sequencing data

The raw 16S rRNA gene sequencing reads were demultiplexed, quality-filtered by Trimmomatic and merged by FLASH with the following criteria: (i) the 300 bp reads were truncated at any site receiving an average quality score of <20 over a 50 bp sliding window, and the truncated reads shorter than 50 bp were discarded, reads containing ambiguous characters were also discarded; (ii) only overlapping sequences longer than 10 bp were assembled according to their overlapped sequence. The maximum mismatch ratio of overlap region is 0.2. Reads that could not be assembled were discarded; (iii) Samples were distinguished according to the barcode and primers, and the sequence direction was adjusted, exact barcode matching, 2 nucleotide mismatch in primer matching.

Operational taxonomic units (OTUs) with 97% similarity cutoff (Liu et al., 2017) were clustered using UPARSE (version 7.1, http://drive5.com/uparse/), and chimeric sequences were identified and removed. The taxonomy of each OTU representative sequence was analyzed by RDP Classifier (http://rdp.cme.msu.edu/) against the 16S rRNA database using confidence threshold of 0.7. Alpha diversity of microbial community was observed through the calculation of chao, Shannon, ace, simpson and coverage index. The beta diversity was analyzed by PCA and hierarchical cluster tree based on the bray-distance to the microbial community. Microbial community composition was illustrated by Venn diagram and bar plot based on the relative abundance. The biomarkers were identified through the linear discriminant analysis (LDA) effect size. The association between the microbial community with environmental factors was evaluated through RDA and two-way correlation network analysis. Microbial phenotype was predicted by BugeBase.

**Supplementary Table S1.** Formulations of growth media for bacteria isolation

| Growth medium | | Formulations |
| --- | --- | --- |
| Nutrient Agar (NA) | Pepton 10 g, Beef extract 3 g, NaCl 5 g, Agar 15 g, Distilled water 1L. | |
| Luria-Bertani (LB) | Tryptone 10 g, Yeast extract 5 g, NaCl 10 g, Agar 15 g, Distilled water 1L. | |
| Reasoner’s 2A Agar (R2A) | Tryptone 0.25 g, Casein hydrolysate 0.5 g, Yeast extract 0.5 g, Soluble starch 0.5 g, K_2_HPO_4_ 0.3 g, MgSO_4_ 0.1 g, Sodium pyruvate 0.3 g, Pepton 0.25 g, Glucose 0.5 g, Agar 15 g, Distilled water 1L. | |
| Triphenytetrazolium Chloride (TTC) | Pepton 10 g, Beef extract 3 g, NaCl 5 g, Agar 15 g, TTC 0.01g, Distilled water 1L. | |
| Plate Count Agar (PCA) | Tryptone 0.25 g, Yeast extract 0.5 g, Glucose 1.0 g, Agar 15 g, Distilled water 1L. | |
| Yeast Peptone Dextrose （YPD） | Pepton 20 g, Glucose 20 g, Yeast extract 10 g, Agar 15 g, Distilled water 1L. | |
| Ashby Glucose (Ashby) | Glucose 10 g, KH_2_PO_4_ 0.2 g, MgSO_4_·7H_2_O 0.2 g, NaCl 0.2 g, CaSO_4_·2H_2_O 0.1 g, CaCO_3_ 5g, Agar 20 g, Distilled water 1L. | |

**Supplementary** **Table S2**. Grading of soil pH, organic matter and major nutrients contents in blueberry orchards

| Grade | pH^1^ | Organic matter^1^  (g/kg) | Total N^2^  (g/kg) | Available N^2^  (mg/kg) | Available P^2^ (mg/kg) | Available K^2^ (mg/kg) |
| --- | --- | --- | --- | --- | --- | --- |
| 1 | 4.5-5.5 | >50 | >2.0 | >150 | >40 | >200 |
| 2 | 4.0-4.5 / 5.5-6.0 | 30.0-50.0 | 1.5-2.0 | 120-150 | 20-40 | 150-200 |
| 3 | 3.5-4.0 | 20.0-30.0 | 1.0-1.5 | 90-120 | 10-20 | 100-150 |
| 4 | ≤3.5 | 10.0-20.0 | 0.75-1.0 | 60-90 | 5-10 | 50-100 |
| 5 | ≥6.0 | <10.0 | 0.5-0.75 | 30-60 | 3-5 | 30-50 |
| 6 |  |  | <0.5 | <30 | <3 | <30 |

Note: Grade 1, 2 and 5 in the table represent the most suitable, suitable and unsuitable soil pH and organic matter content, respectively. Simultaneously, grade 1 - 6 represent very high, high, medium, low, very low and dramatically low level of major nutrients.

^1^Dong, K. F., Liu, F. C. and Jiang, H. T. (2016). Analysis of pH value and primary nutrients in blueberry garden soil. *Chinese Agriculture Science Bulletin*. 32, 141-145. doi: 10.11924/j.issn.1000-6850.casb15070084

^2^The second national Soil Survey of China, 1994.

**Supplementary Table S3**. Rhizosphere common species of healthy old tree (O) and young seedlings (OG), and poor growth seedlings (OB).

| OTU | Phylum | Order | Genus | O | OG | OB |
| --- | --- | --- | --- | --- | --- | --- |
| OTU2002 | Proteobacteria | Xanthomonadales | Chujaibacter | 1936 | 557 | 1 |
| OTU2209 | Actinobacteriota | Frankiales | Acidothermus | 1734 | 54 | 101 |
| OTU2411 | Actinobacteriota | Gaiellales | norank_f__norank_Gaiellales | 1251 | 170 | 131 |
| OTU4379 | Actinobacteriota | Micrococcales | Intrasporangium | 4 | 1234 | 67 |
| OTU4774 | Firmicutes | Bacillales | Sporosarcina | 94 | 1187 | 7 |
| OTU4423 | Actinobacteriota | Micrococcales | unclassified_f__Microbacteriaceae | 26 | 1073 | 25 |
| OTU1765 | Actinobacteriota | Frankiales | Acidothermus | 909 | 235 | 46 |
| OTU2907 | Proteobacteria | Rhizobiales | Bradyrhizobium | 410 | 239 | 261 |
| OTU2112 | Firmicutes | Bacillales | Bacillus | 698 | 248 | 30 |
| OTU4541 | Proteobacteria | Sphingomonadales | Sphingomonas | 15 | 645 | 178 |
| OTU795 | Proteobacteria | Rhizobiales | Pseudolabrys | 82 | 18 | 598 |
| OTU4113 | Firmicutes | Bacillales | Sporosarcina | 81 | 638 | 6 |
| OTU4376 | Actinobacteriota | Frankiales | Blastococcus | 2 | 612 | 7 |
| OTU2145 | Chloroflexi | Ktedonobacterales | norank_f__JG30-KF-AS9 | 734 | 66 | 13 |
| OTU2446 | Proteobacteria | Gammaproteobacteria_Incertae_Sedis | Acidibacter | 749 | 9 | 3 |
| OTU4543 | Firmicutes | Bacillales | Bacillus | 40 | 567 | 1 |
| OTU2299 | Proteobacteria | Acetobacterales | unclassified_f__Acetobacteraceae | 578 | 79 | 13 |
| OTU2139 | Proteobacteria | Elsterales | norank_f__norank_o__Elsterales | 687 | 1 | 6 |

**Supplementary** **Table S4.** Rhizosphere unique species of poor growth seedlings (OB).

| OTU | Phylum | Order | Genus | OB |
| --- | --- | --- | --- | --- |
| OTU4008 | Latescibacterota | norank_c__norank_p__Latescibacterota | norank_f__norank_o__norank_c__norank_p__Latescibacterota | 502 |
| OTU3981 | Proteobacteria | Dongiales | Dongia | 208 |
| OTU3271 | Acidobacteriota | Vicinamibacterales | norank_f__Vicinamibacteraceae | 156 |
| OTU3224 | Acidobacteriota | Pyrinomonadales | RB41 | 155 |
| OTU3678 | Acidobacteriota | Vicinamibacterales | norank_f__norank_o__Vicinamibacterales | 154 |
| OTU1112 | Methylomirabilota | Rokubacteriales | norank_f__norank_o__Rokubacteriales | 138 |
| OTU2941 | Acidobacteriota | norank_c__Subgroup_5 | norank_f__norank_o__norank_c__Subgroup_5 | 123 |
| OTU3113 | Chloroflexi | SBR1031 | norank_f__norank_o__SBR1031 | 127 |
| OTU981 | Chloroflexi | Anaerolineales | norank_f__Anaerolineaceae | 123 |

**Supplementary** **Table S5.** Nitrogen fixing and phosphorus solubilizing ability assessment of the isolates.

| Isolates | Systematic identification | Culture Medium | | | |
| --- | --- | --- | --- | --- | --- |
|  |  | Ashby | Pikovskaya（inorganic P） | Pikovskaya （organic P） |  |
| O_Ashby_1 | Bacillus megaterium (98.0%) | ++ | +++ | - |  |
| O_Ashby_2 | Bacillus megaterium (100%) | ++ | +++ | - |  |
| O_Ashby_3 | Bacillus megaterium (100%) | ++ | - | +++ |  |
| O_Ashby_4 | Bacillus megaterium (100%) | ++ | - | +++ |  |
| O_Ashby_5 | Bacillus megaterium (100%) | ++ | - | +++ |  |
| O_Ashby_6 | Microbacterium hydrocarbonoxydans (99.4%) | ++ | - | +++ |  |
| O_LB_1 | Bacillus cereus (100%) | **++** | **+++** | **+++** |  |
| O_LB_2 | Bacillus megaterium (99.9%) | **++** | **+++** | **+++** |  |
| O_LB_3 | Lysinibacillus xylanilyticus (99.9%) | - | - | - |  |
| O_LB_4 | Bacillus megaterium (99.9%) | ++ | +++ | - |  |
| O_LB_5 | Bacillus megaterium (99.9%) | ++ | - | +++ |  |
| O_LB_6 | Bacillus megaterium (100%) | + | - | +++ |  |
| O_LB_7 | Bacillus aryabhattai (100%) | + | +++ | +++ |  |
| O_LB_8 | Lysinibacillus mangiferihumi (99.8%) | - | - | - |  |
| O_LB_9 | Bacillus cereus (99.9%) | + | - | - |  |
| O_LB_10 | Microbacterium paraoxydans (99.9%) | - | ++++ | - |  |
| O_NA_1 | Bacillus sphaericus (99.9%) | - | - | +++ |  |
| O_NA_2 | Bacillus sphaericus (99.9%) | - | - | + |  |
| O_NA_3 | Bacillus sphaericus (99.9%) | - | - | - |  |
| O_NA_4 | Bacillus humi (99.6%) | - | - | - |  |
| O_NA_5 | Bacillus sphaericus (99.9%) | - | - | + |  |
| O_NA_6 | Lysinibacillus sphaericus (99.9%) | ++ | - | - |  |
| O_NA_7 | Bacillus niacini (99.9%) | - | - | +++ |  |
| O_NA_8 | Bacillus niacini (99.9%) | + | - | +++ |  |
| O_NA_9 | Bacillus sphaericus (99.8%) | - | - | - |  |
| O_NA_10 | Bacillus sphaericus (99.9%) | - | - | - |  |
| O_NA_11 | Bacillus oleronius (99.7%) | - | - | - |  |
| O_NA_12 | Lysinibacillus xylanilyticus (99.9%) | - | +++ | - |  |
| O_NA_13 | Bacillus aryabhattai (100%) | ++ | ++ | +++ |  |
| O_PCA_1 | Bacillus cereus (100%) | + | - | - |  |
| O_PCA_2 | Bacillus megaterium (100%) | - | ++ | - |  |
| O_PCA_3 | Bacillus pseudomycoides (100%) | - | - | +++ |  |
| O_PCA_4 | Bacillus sphaericus (99.8%) | - | - | +++ |  |
| O_PCA_5 | Bacillus cereus (99.0%) | - | - | - |  |
| O_PCA_6 | Bacillus drentensis (100%); | - | ++ | + |  |
| O_PCA_7 | Bacillus megaterium (100%) | + | +++ | ++ |  |
| O_PCA_8 | Bacillus megaterium (100%) | ++ | +++ | ++ |  |
| O_PCA_9 | Bacillus sphaericus (99.9%) | - | - | - |  |
| O_PCA_10 | Bacillus sphaericus (99.8%) | - | - | - |  |
| O_PCA_11 | Bacillus sphaericus (99.9%) | - | - | - |  |
| O_PCA_12 | Bacillus sphaericus (99.9%) | - | - | + |  |
| O_PCA_13 | Bacillus oleronius (99.0%) | ++ | +++ | ++ |  |
| O_PCA_14 | Bacillus proteolyticus (100%) | ++ | +++ | - |  |
| O_PCA_15 | Bacillus sphaericus (99.9%) | - | - | + |  |
| O_PCA_16 | Bacillus sphaericus (99.9%) | - | - | ++ |  |
| O_R2A_1 | Bacillus sphaericus (99.7%) | + | - | + |  |
| O_R2A_2 | Brevibacillus laterosporus (99.9%); | - | - | - |  |
| O_R2A_3 | Lysinibacillus sphaericus (99.9%) | - | + | - |  |
| O_R2A_4 | Lysinibacillus xylanilyticus (99.9%) | - | - | ++ |  |
| O_R2A_5 | Bacillus siamensis (100%) | ++ | +++ | ++ |  |
| O_R2A_6 | Bacillus oleronius (99.8%) | - | - | - |  |
| O_R2A_7 | Lysinibacillus xylanilyticus (99.9%) | - | - | +++ |  |
| O_R2A_8 | Bacillus sphaericus(99.9%) | + | + | - |  |
| O_R2A_9 | Bacillus niacini(99.7%) | - | +++ | - |  |
| O_R2A_10 | Bacillus pseudomycoides(100%) | - | - | - |  |
| O_R2A_11 | Bacillus megaterium (100%) | ++ | +++ | + |  |
| O_TTC_1 | Citrobacter amalonaticus (99.8%) | - | ++ | +++ |  |
| O_TTC_2 | Bacillus sphaericus (99.8%) | - | - | +++ |  |
| O_TTC_3 | Bacillus sphaericus (99.8%) | - | - | - |  |
| O_TTC_4 | Bacillus sphaericus (99.8%) | - | - | - |  |
| O_TTC_5 | Bacillus oleronius (100%) | - | - | +++ |  |
| O_TTC_6 | Bacillus sphaericus (99.9%) | - | - | - |  |
| O_TTC_7 | Bacillus aryabhattai (100%) | ++ | +++ | ++ |  |
| O_TTC_8 | Bacillus sphaericus (99.9%) | - | - | - |  |
| O_TTC_9 | Bacillus megaterium (99.9%) | + | +++ | - |  |
| O_TTC_10 | Bacillus sphaericus (99.8%) | - | - | - |  |
| O_TTC_11 | Bacillus oleronius (99.9%) | - | - | - |  |
| O_TTC_12 | Bacillus oleronius (99.9%) | ++ | + | +++ |  |
| O_YPD_1 | Bacillus megaterium (100%) | - | - | - |  |
| O_YPD_2 | Bacillus sphaericus (99.8%) | **-** | **-** | **-** |  |
| O_YPD_3 | Bacillus sphaericus (99.8%) | - | - | - |  |
| O_YPD_4 | Bacillus sphaericus (99.8%) | - | - | +++ |  |
| O_YPD_5 | Bacillus velezensis (99.9%) | + | +++ | +++ |  |
| O_YPD_6 | Bacillus oleronius (99.7%) | **++** | **+++** | **+++** |  |
| O_YPD_7 | Bacillus sphaericus (99.9%) | ++ | - | - |  |
| O_YPD_8 | Bacillus sphaericus (99.8%) | - | - | + |  |
| O_YPD_9 | Bacillus sphaericus (99.8%) | - | - | - |  |
| O_YPD_10 | Bacillus megaterium (100%) | - | - | - |  |
| O_YPD_11 | Lysinibacillus xylanilyticus (99.9%) | - | - | - |  |
| O_YPD_12 | Lysinibacillus xylanilyticus (99.9%) | - | - | - |  |

Note: The symbol “-” represents the isolated bacterium cannot grow on the solid culture medium. Symbol “+” represents the isolated bacterium can grow on the medium. Symbol “++” represents the isolated bacterium growing well but no clear zone appearing on the medium. Symbol “+++” represents the isolated bacterium growing well and clear zone appeared on the medium. Symbol “++++” represents the clear zone was spread on the medium.

**Supplementary Figure S1.** Microbial community of O’Neal blueberry rhizosphere. (A) Venn diagram; (B) rhizosphere common species of healthy old tree (O) and young seedlings (OG), and poor growth seedlings (OB); (C) rhizosphere unique species of poor growth seedlings (OB).

**Supplementary Figure S2.** Rhizosphere bacteria difference among O’Neal blueberry of healthy old tree (O) and young seedlings (OG), and poor growth seedlings (OB).


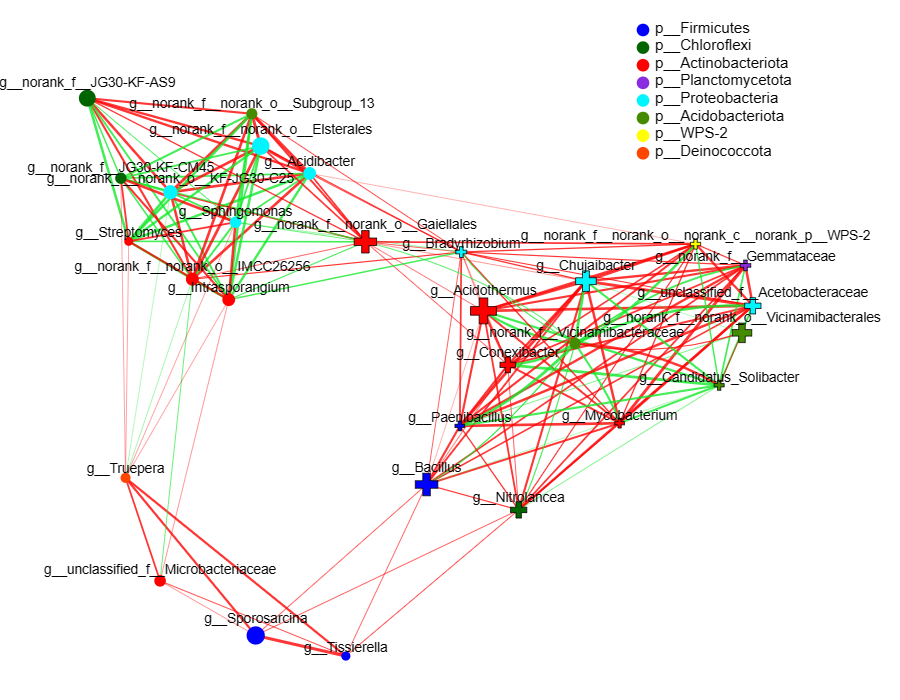


**Supplementary Figure S3.** One-way correlation network of blueberry rhizobacterial.
